# Supplementary material for: Quinoline- and Isoindoline-Integrated Polycyclic Compounds as Antioxidant, and Antidiabetic Agents Targeting the Dual Inhibition of α-Glycosidase and α-Amylase Enzymes
Source: Pharmaceuticals (Basel). 2023 Aug 30;16(9):1222. doi: 10.3390/ph16091222 (PMC10535292; doi:10.3390/ph16091222)

Supplementary file

Quinoline and Isoindoline Integrated Polycyclic Compounds as Antioxidant, and Antidiabetic Agents Targeting Dual Inhibition of α-glycosidase and α-amylase enzymes

Mohammed Al-Ghorbani[1]*, Osama Alharbi[1], Abdel-Basit Al-Odayni[2], Naaser A.Y. Abduh[3]

[1] Department of Chemistry, College of Science and Arts, Ulla, Taibah University, Medina Manora, Saudi Arabia; mghorbani@taibahu.edu.sa oamharbi@taibahu.edu.sa
[2] Department of Restorative Dental Science, College of Dentistry, King Saud University, P. O. Box 60169, Riyadh 11545, Saudi Arabia; aalodayni@ksu.edu.sa
[3] Department of Chemistry, College of Science, King Saud University, P. O. Box 2455, Riyadh 11451, Saudi Arabia; 439106262@student.ksu.edu.sa

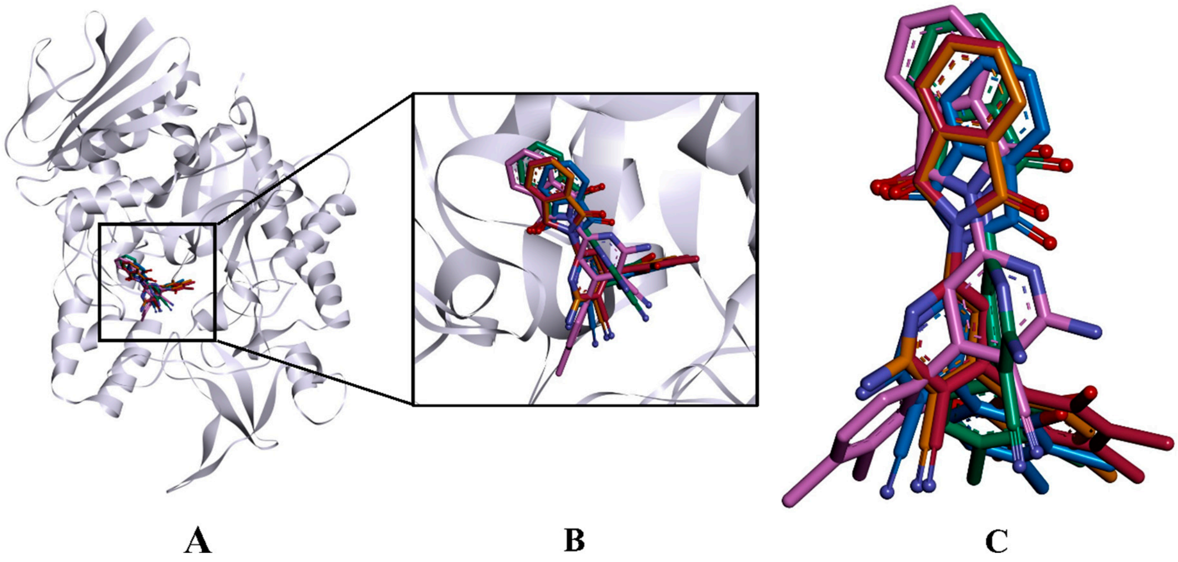

Figure S1: Snapshots from the MD simulation of compound **7d** with α-glucosidase at different time intervals. A) ligand 7d bound conformations inside the inhibitor binding site; B) Arrangement of the binding conformations; and C) ligand-only conformations of 7d. Red: 20 ns, Orange: 40 ns, Blue: 60 ns, Magenta: 80 ns, and Green: 100 ns.

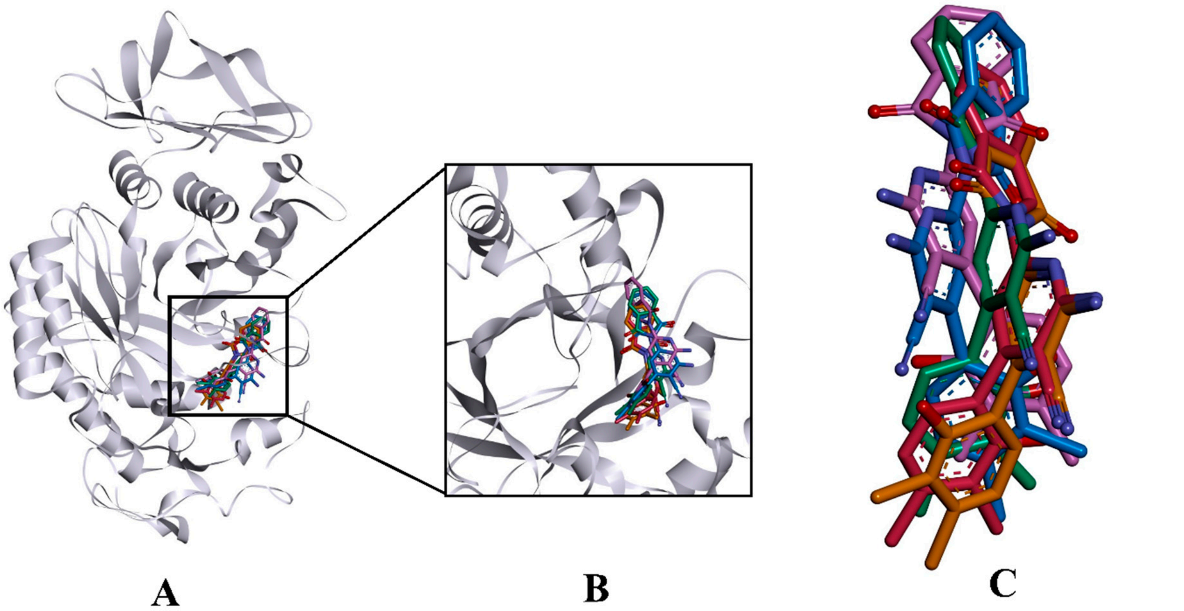

Figure S2: Snapshots from the MD simulation of compound **7d** with α-amylase at different time intervals. A) ligand 7d bound conformations inside the inhibitor binding site; B) Arrangement of

the binding conformations; and C) ligand-only conformations of 7d. Red: 20 ns, Orange: 40 ns, Blue: 60 ns, Magenta: 80 ns, and Green: 100 ns.

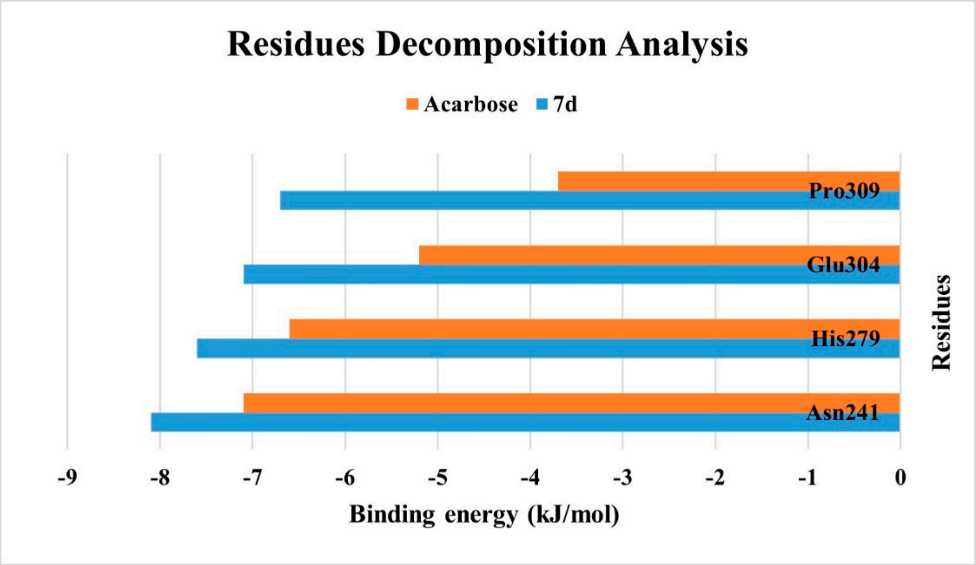

Figure S3: The interactive amino acid residues decomposition analysis of α-glucosidase with compound 7d and acarbose. The binding energy (kJ/mol) contribution of Pro309, Glu304, His279, and Asn24 in α-glucosidase-compound 7d complex estimated from 5000 frames (50 ns) of MD simulation.

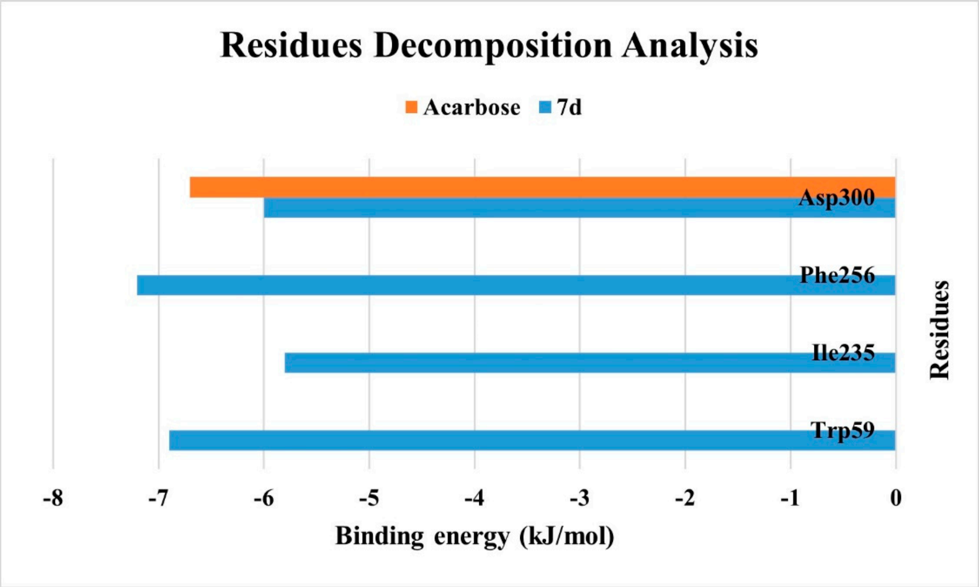

Figure S4: The interactive amino acid residues decomposition analysis of α-amylase with compound 7d and acarbose. The binding energy (kJ/mol) contribution of Asp300, Phe256, Ile235, and Trp59 in α-amylase-compound 7d complex estimated from 5000 frames (50 ns) of MD simulation.

Figures S5: NMR and Mass for the target compounds

^{1}H NMR of *N′*-(1-(4-(1,3-dioxoisoindolin-2-yl)phenyl)ethylidene)-2-(quinolin-8-yloxy)acetohydrazide 3.

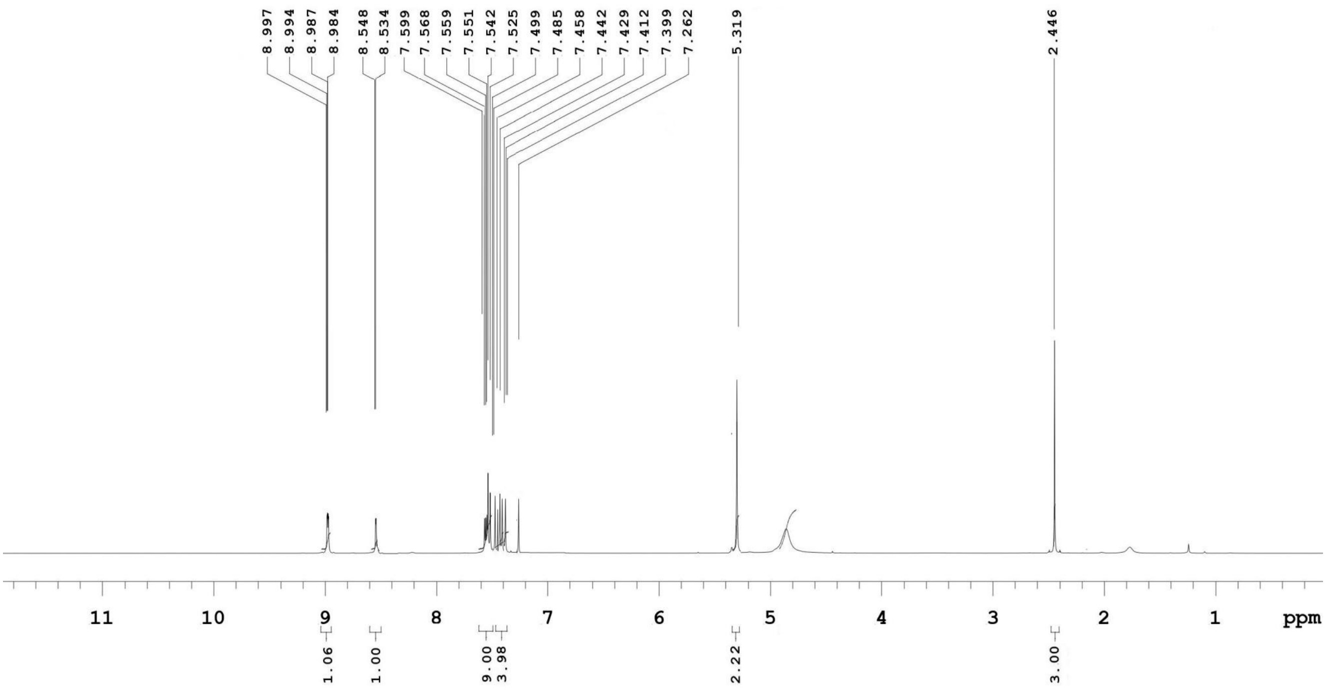

^{13}C NMR of *N′*-(1-(4-(1,3-dioxoisoindolin-2-yl)phenyl)ethylidene)-2-(quinolin-8-yloxy)acetohydrazide 3.

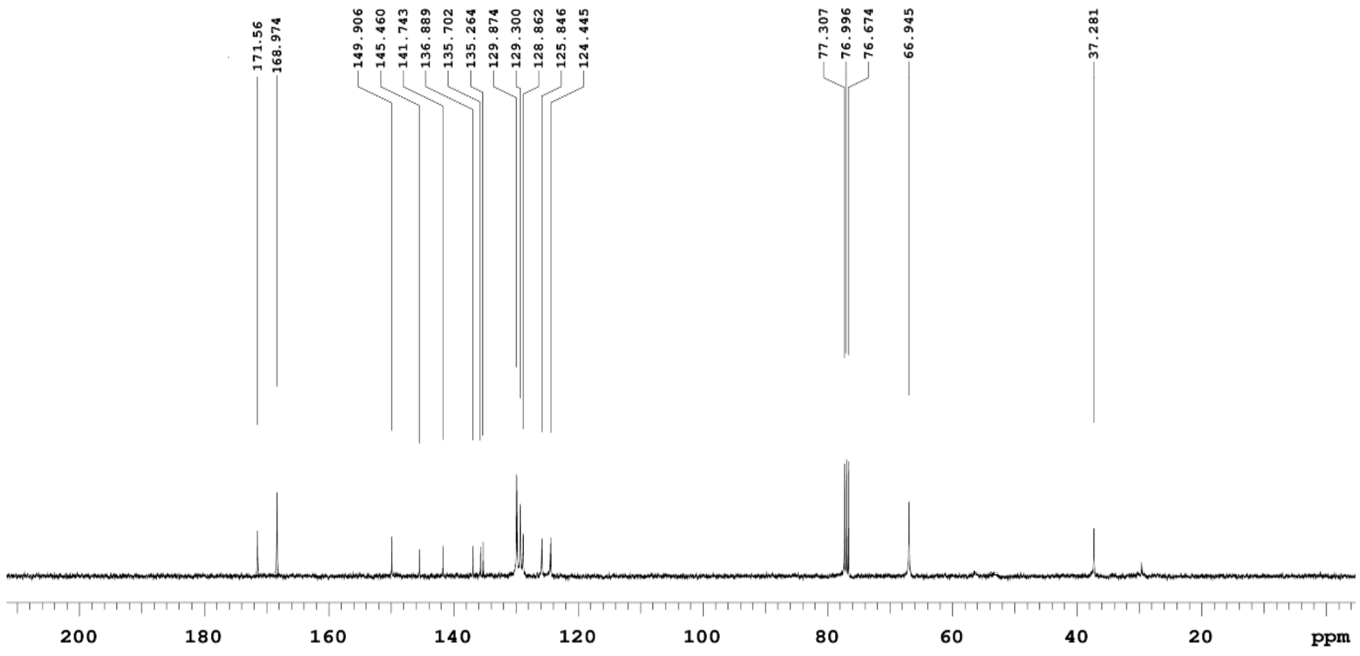

Mass spectrum of *N′*-(1-(4-(1,3-dioxoisoindolin-2-yl)phenyl)ethylidene)-2-(quinolin-8-yloxy)acetohydrazide 3.

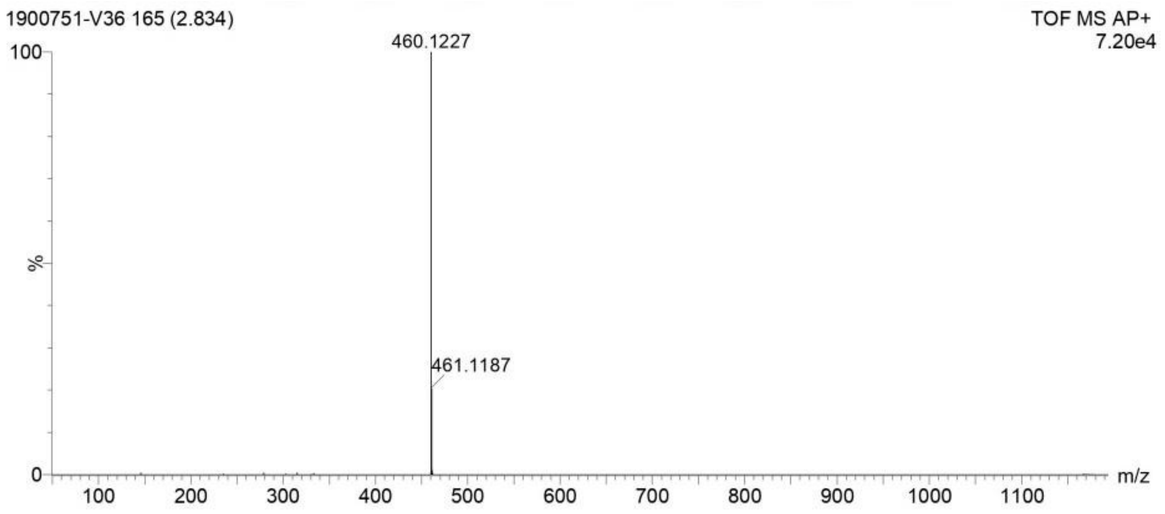

^{1}H NMR of 5-Amino-3-(4-chlorophenyl)-1-(2-(quinolin-8-yloxy)acetyl)-1*H*-pyrazole-4-carbonitrile 4b.

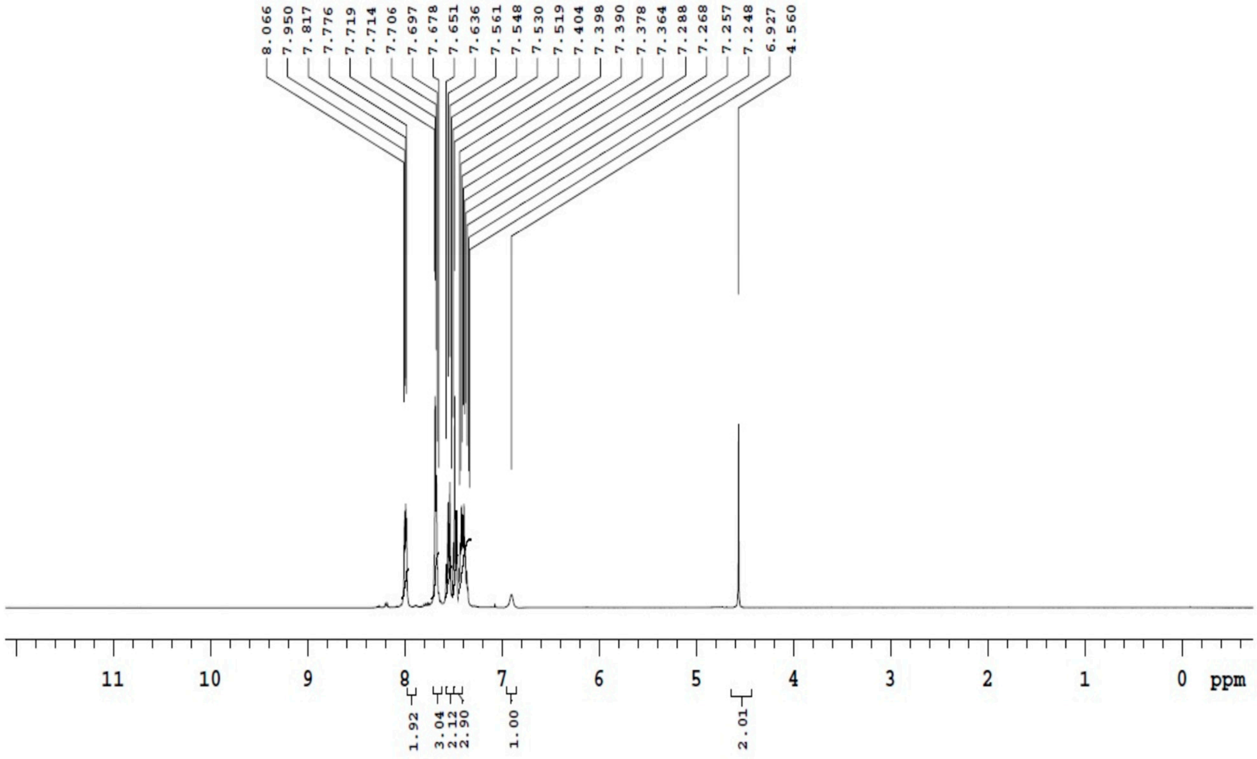

^{13}C NMR of 5-Amino-3-(4-chlorophenyl)-1-(2-(quinolin-8-yloxy)acetyl)-1*H*-pyrazole-4-carbonitrile 4b.

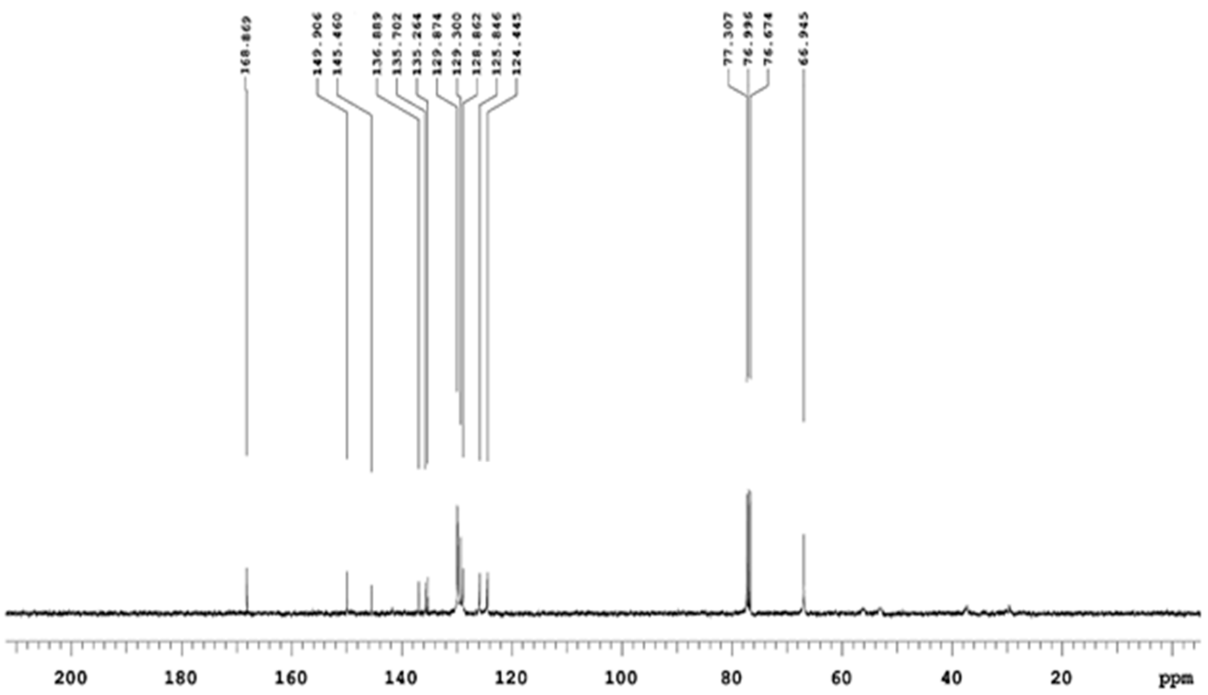

Mass spectrum of 5-Amino-3-(4-chlorophenyl)-1-(2-(quinolin-8-yloxy)acetyl)-1*H*-pyrazole-4-carbonitrile 4b.

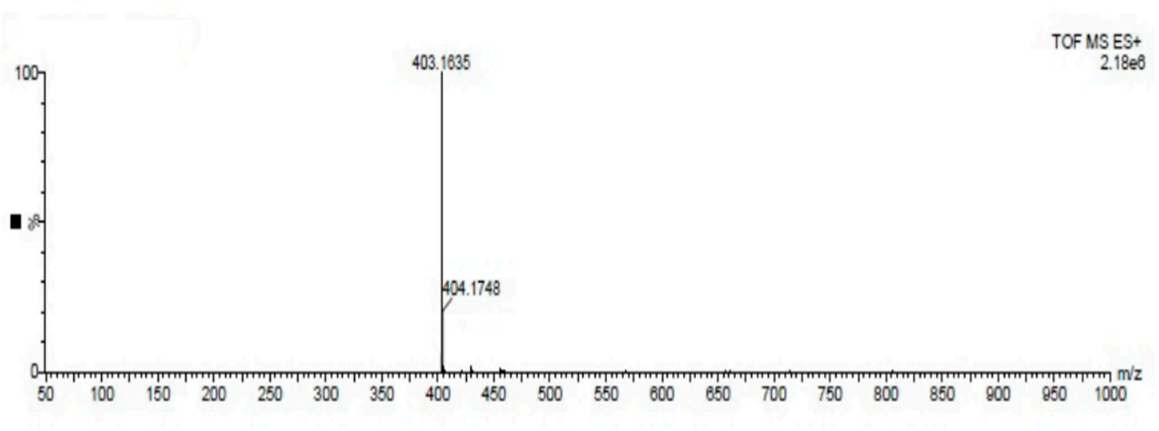

^{1}H NMR of 5-((quinolin-8-yloxy)methyl)-3*H*-1,2,4-triazole-3-thione 6.

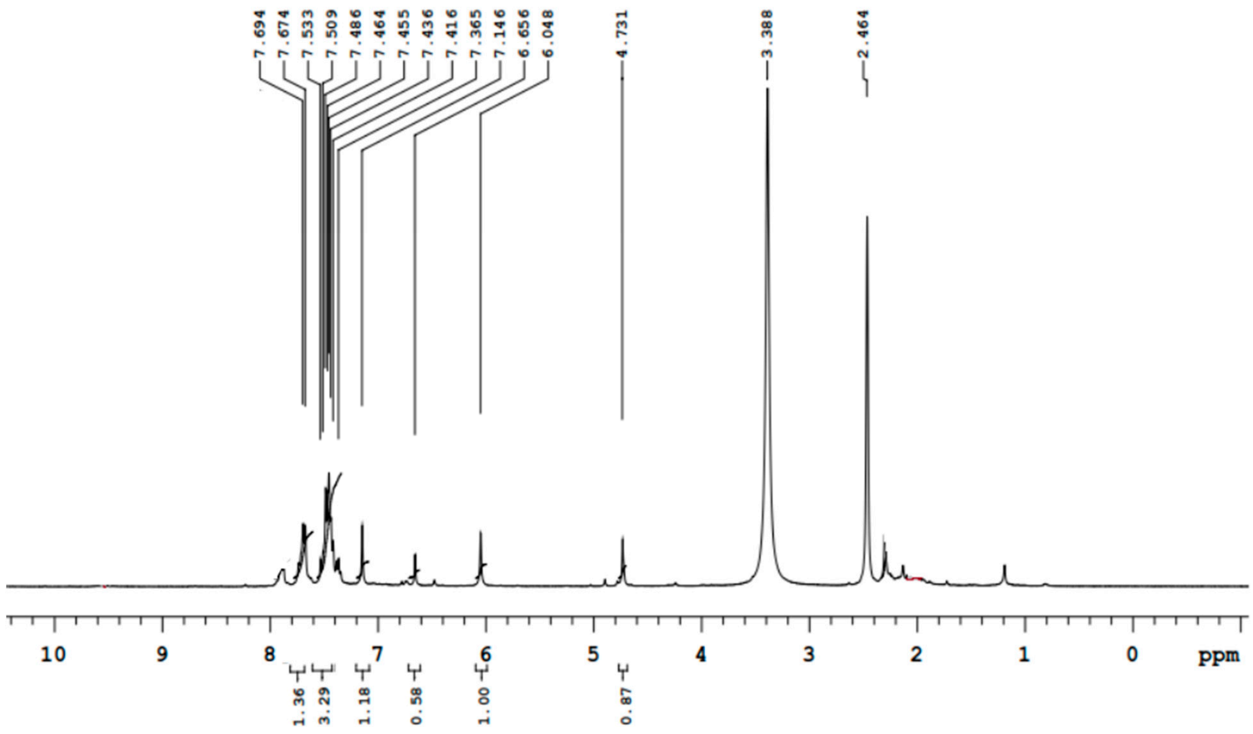

^{13}C NMR of 5-((quinolin-8-yloxy)methyl)-3*H*-1,2,4-triazole-3-thione 6.

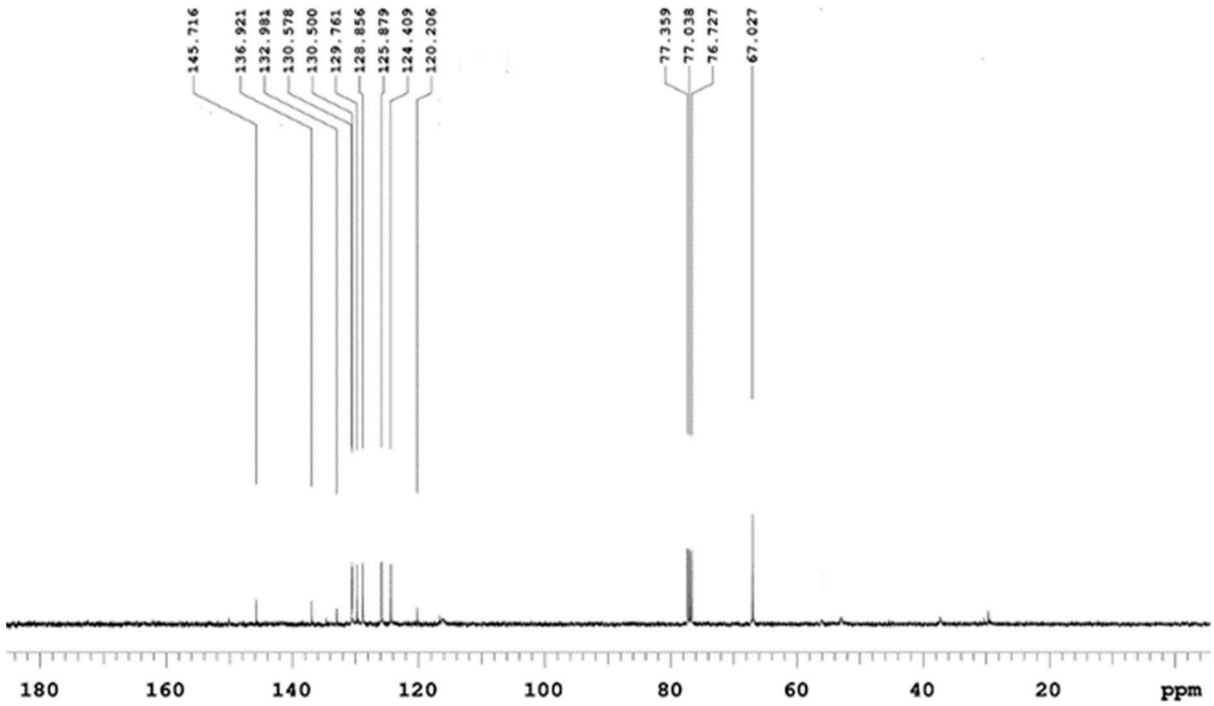

^{1}H NMR of 2-amino-6-(1,3-dioxoisoindolin-2-yl)-4-phenylnicotinonitrile 7a.

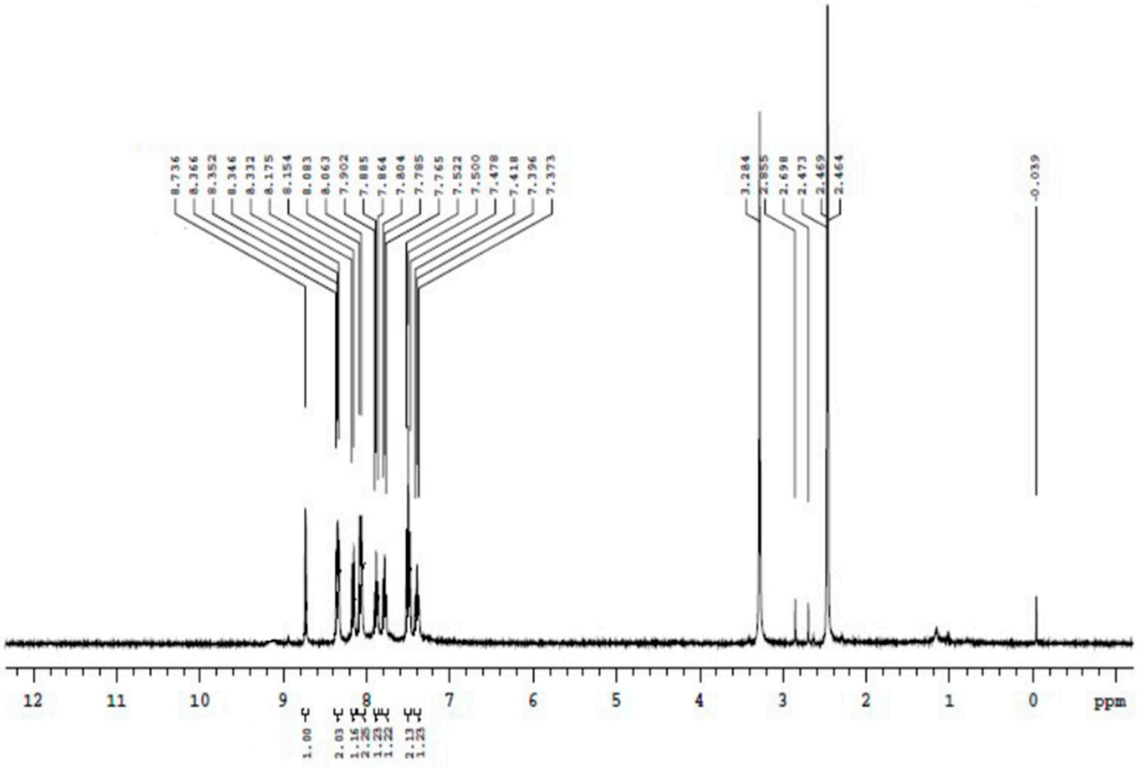

^{13}C NMR of 2-amino-6-(1,3-dioxoisoindolin-2-yl)-4-phenylnicotinonitrile 7a.

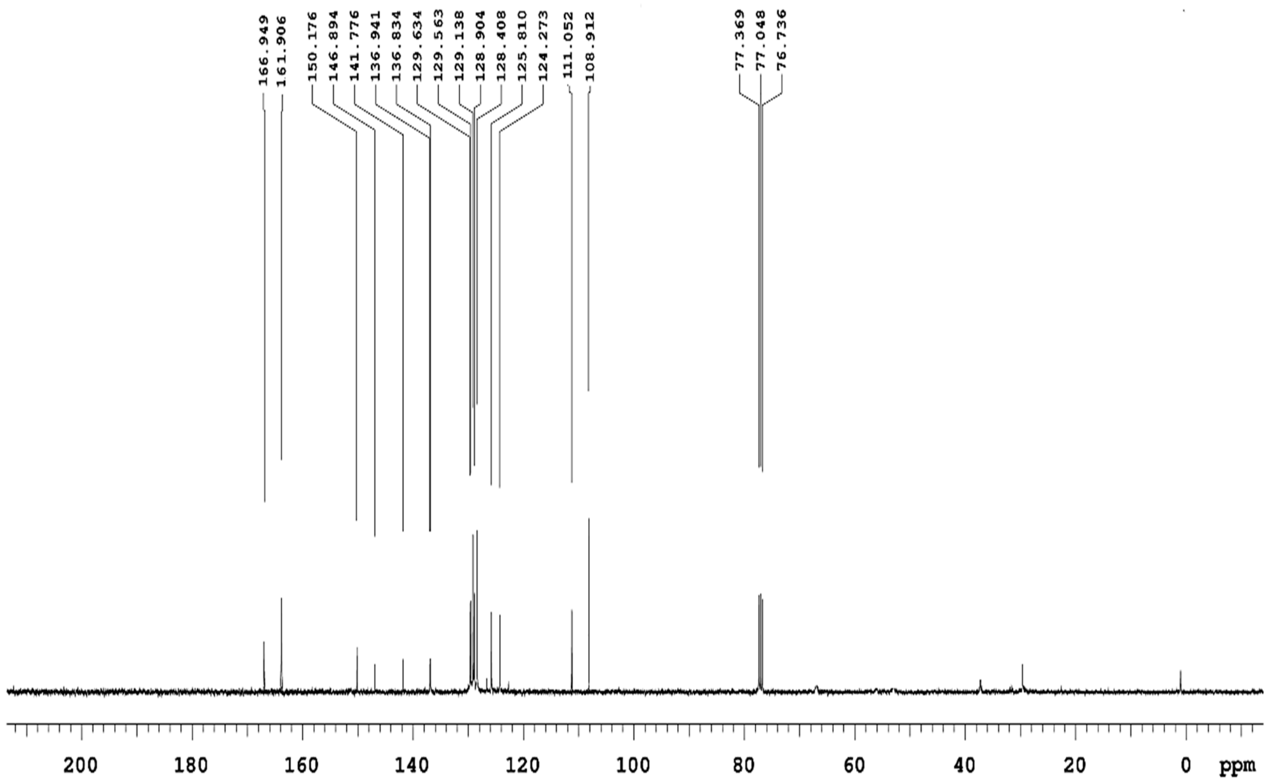

Mass spectrum of 2-amino-6-(1,3-dioxoisoindolin-2-yl)-4-phenylnicotinonitrile 7a.

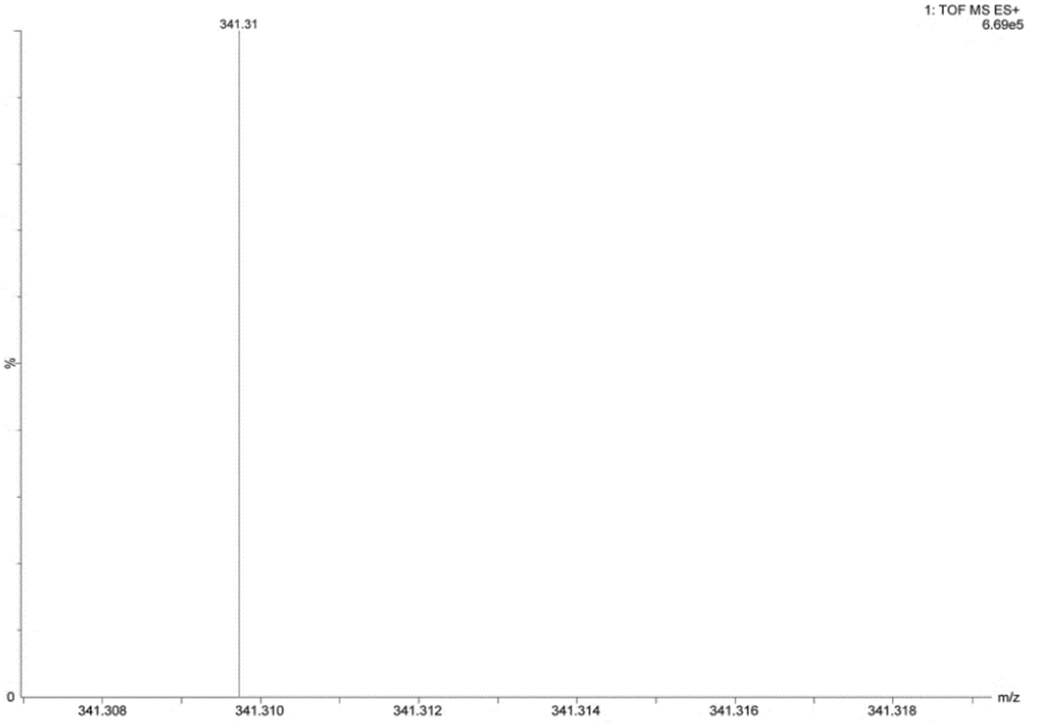

^{1}H NMR of 6-(1,3-dioxoisoindolin-2-yl)-2-oxo-4-phenyl-1,2-dihydropyridine-3-carbonitrile 8a.

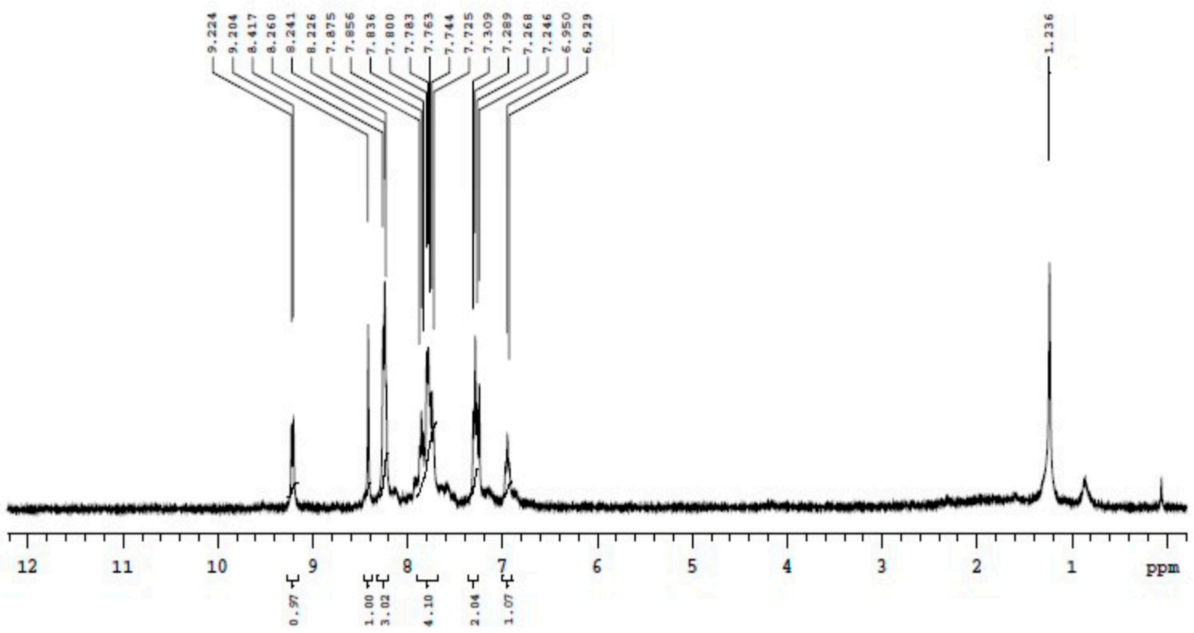

^{13}C NMR of 6-(1,3-dioxoisoindolin-2-yl)-2-oxo-4-phenyl-1,2-dihydropyridine-3-carbonitrile 8a.

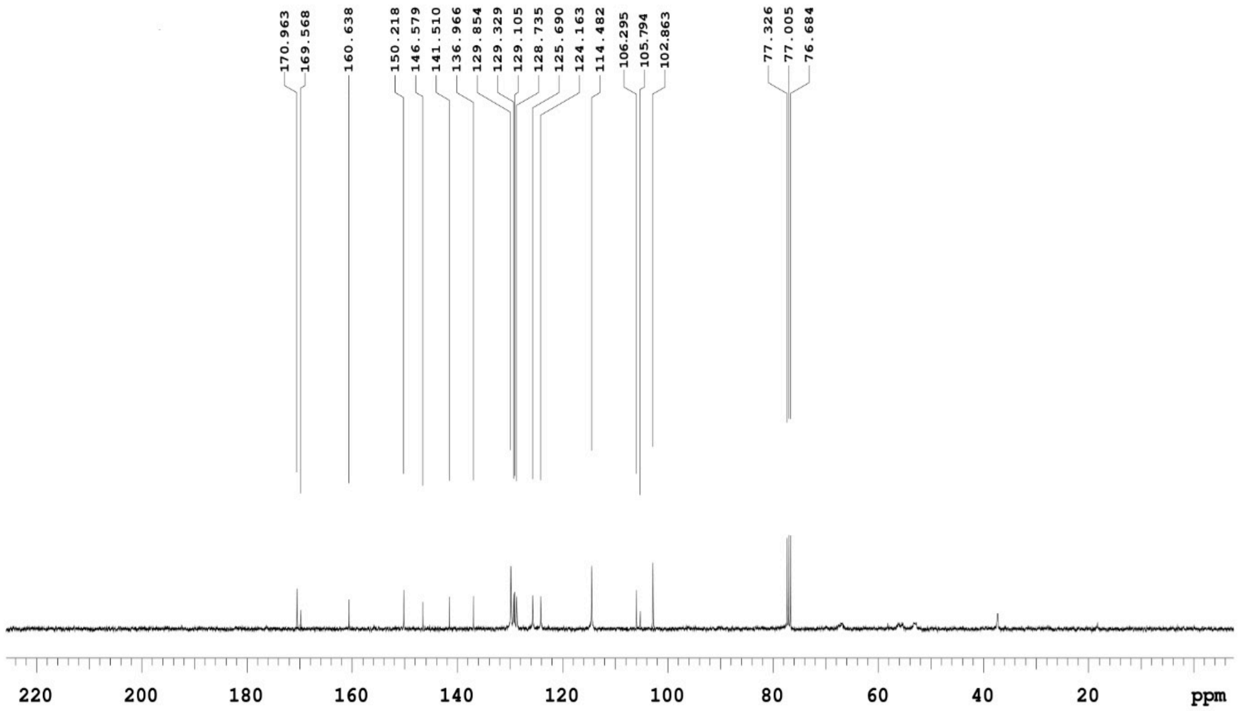

Mass spectrum of 6-(1,3-dioxoisoindolin-2-yl)-2-oxo-4-phenyl-1,2-dihydropyridine-3-carbonitrile 8a.

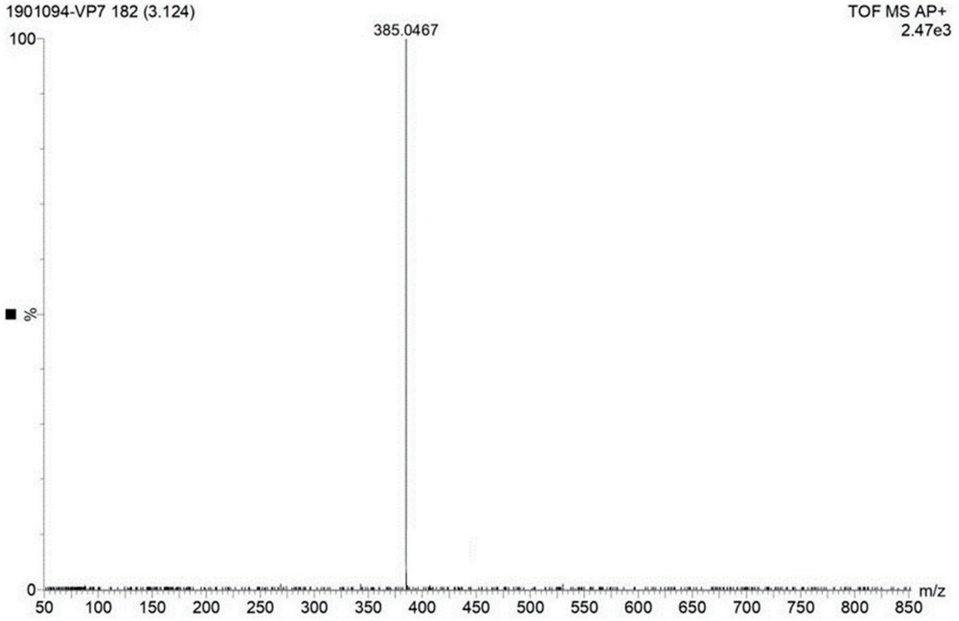

^{1}H NMR of 8-(2-((2-(1-(4-(1H-Tetrazol-1-yl)phenyl)ethylidene)hydrazinyl)oxy)-2-oxoethoxy)quinoline 2

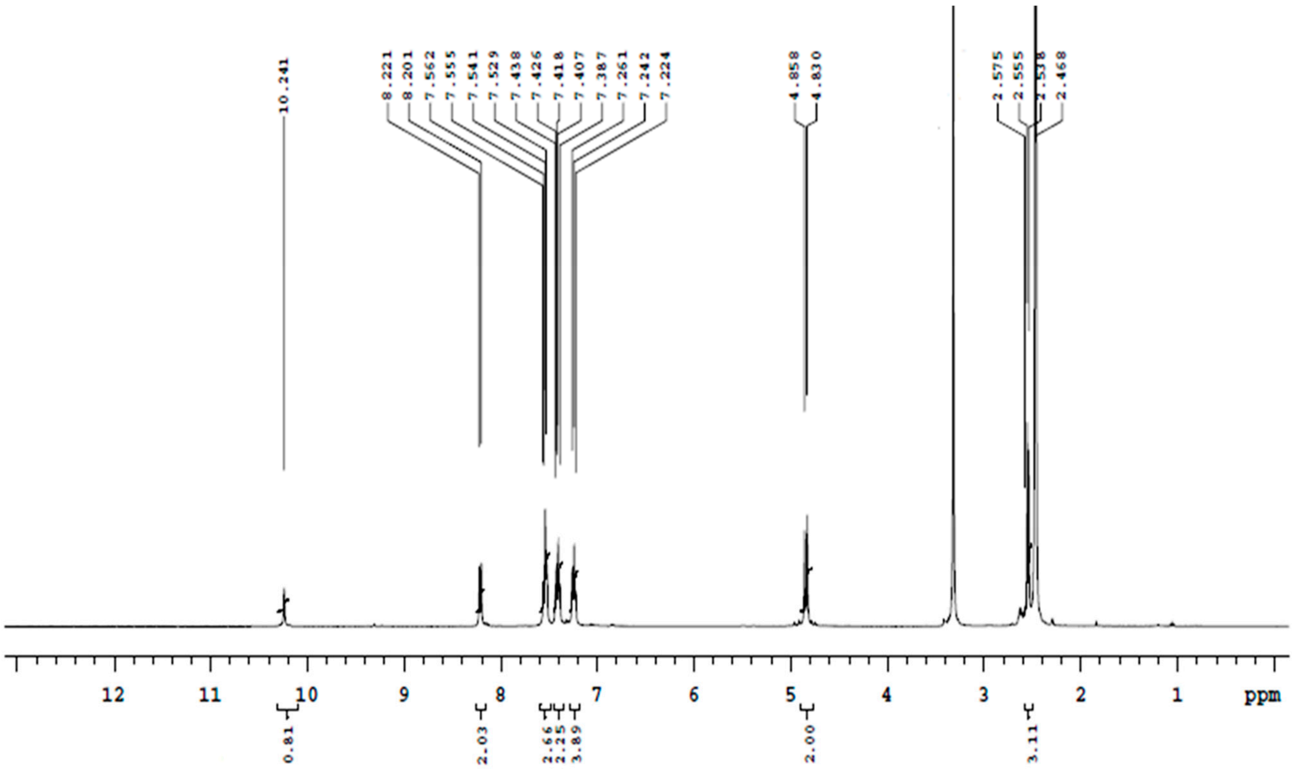

^{1}H NMR of 5-Amino-3-(4-nitrophenyl)-1-(2-(quinolin-8-yloxy)acetyl)-1H-pyrazole-4-carbonitrile 4a

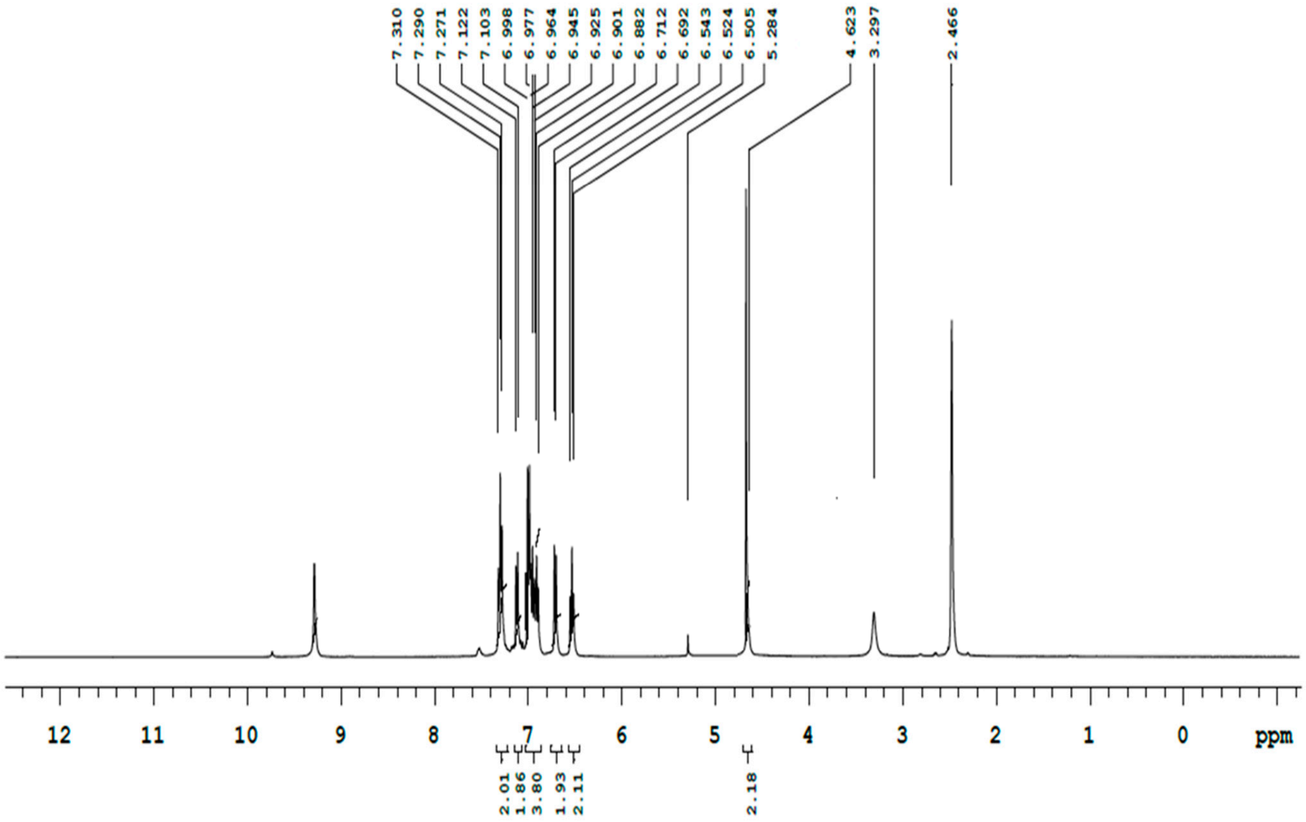

^{1}H NMR for 6-(1,3-Dioxoisoindolin-2-yl)-4-(4-nitrophenyl)-2-oxo-1,2-dihydropyridine-3-carbonitrile 8b

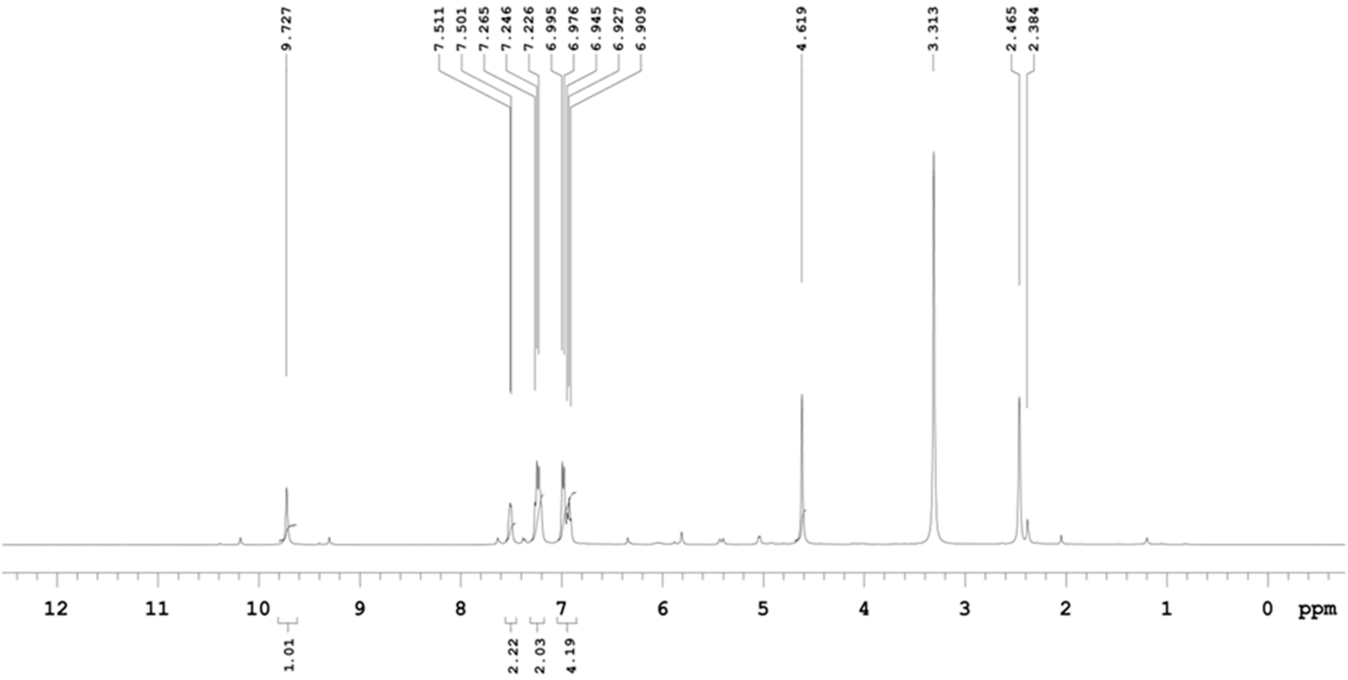

Supplement: Supplementary file 1 [file pharmaceuticals-16-01222-s001.zip › pharmaceuticals-2537682-supplementary.pdf]
